# Effective treatment of recalcitrant Hailey-Hailey disease with dupilumab

**DOI:** 10.1016/j.jdcr.2024.09.030

**Published:** 2024-11-20

**Authors:** Deep Patel, Jason Rosenberg, Jacob Cohen, Kristen E. Holland

**Affiliations:** a Department of Dermatology, Medical College of Wisconsin, Milwaukee, Wisconsin; b Ascension Columbia St. Mary’s Hospital, Milwaukee, Wisconsin; c University of Michigan, Ann Arbor, Michigan

**Keywords:** benign chronic pemphigus, clinical case report, dupilumab, genetic skin disorder, Hailey-Hailey disease, inflammatory skin disease, monoclonal antibody, recalcitrant skin condition, skin barrier dysfunction, targeted biologic therapy

## Introduction

Hailey-Hailey disease (HHD), also known as benign familial pemphigus, is a rare autosomal dominant genodermatoses that classically presents with intertriginous painful, pruritic plaques, and erosions.^1^ Histologically, the disease is identified by full thickness acantholysis of the spinous layer in a hyperplastic epidermis, resulting in the appearance of a “dilapidated brick wall.”^1^ HHD arises from mutations in the ATP2C1 gene which encodes for calcium pumps in the Golgi apparatus.^1^ The disease typically presents in the flexural areas in individuals between 20 and 40 years of age.^2^ No curative treatment for HHD is available, but common treatments include topical corticosteroids, topical immunomodulators, and antibiotics. We present a patient with bilateral, axillary HHD disease that was recalcitrant to multiple topical and oral therapies that subsequently cleared with dupilumab.

## Case presentation

**Fig 1:**
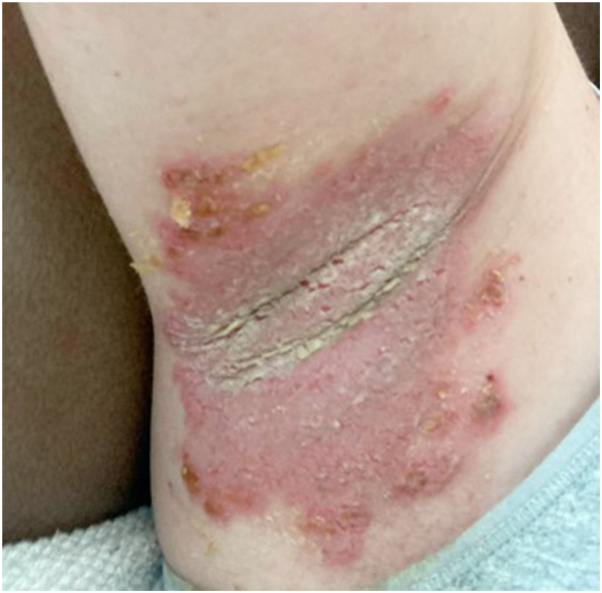
Before starting treatment.

A 36-year-old woman with a history of HHD for more than 20 years historically had mild disease controlled by topical clindamycin solution until her disease flared substantially in October 2021(Fig 1) with erythema, scale, and malodor in her bilateral axilla. Treatment with an ultrapotent topical steroid was initially effective. She had a subsequent flare of disease after laser hair removal of the axillae in August 2022, which was then recalcitrant to ultrahigh potent topical steroids followed by tacrolimus 0.1% ointment. Given the uncharacteristic response to prior treatment, a biopsy was performed for histologic correlation. The biopsy showed prominent intraepidermal acantholysis sparing adnexal epithelium and papillary dermal mixed inflammation, consistent with her long-standing disease. Over the next several months, numerous treatments were trialed with no improvement: calcipotriene ointment, clindamycin solution, high-potency topical steroids, oral azithromycin, and intralesional triamcinolone. It is worth noting that the patient became pregnant during this time. At her office visit in February 2023, safe systemic therapy during third trimester pregnancy was discussed, and oral naltrexone was started.^3^^,^^4^

**Fig 2:**
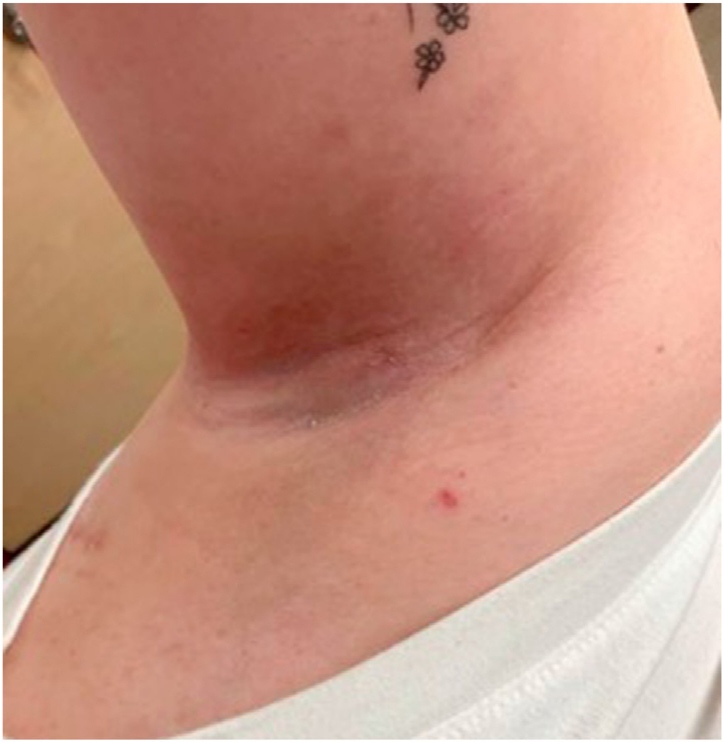
Rapid clearance after initiating treatment.

She continued intermittent high-potency steroids under occlusion, oral naltrexone, and oral antibiotics with intermittent painful flares over the next few months. A comprehensive timeline of treatments and treatment response before starting with the failure of conventional therapy, and emerging literature to support a potential benefit of dupilumab in HHD (Table I), she was started on dupilumab in July 2023 in combination with oral naltrexone. A comprehensive timeline of treatments and treatment response before starting dupilumab is provided in Table II.^5^, ^6^, ^7^, ^8^, ^9^ She rapidly cleared with the addition of dupilumab (Fig 2) and maintained on biweekly injection as monotherapy. She has maintained satisfactory control of her HHD now over 10 months into treatment.

**Table I:** Reports on treatment

Patient age (y)/gender	Duration of HHD	Affected areas	Previous treatments	Dupilumab treatment duration	Concomitant treatments	Outcome	Adverse events	Source
50/F	20 y	Inferior mid-back, bilateral abdomen inframammary folds, inguinal folds, bilateral axillae	Isotretinoin, etanercept, acitretin, prednisone, oral antibiotics, low-dose oral naltrexone, cyclosporine antihistamines, topical corticosteroids, topical clindamycin, silver sulfadiazine, clotrimazole, and topical fluorouracil	21 mo	None	Significant improvement after 2 mo. Reduction in size and thickness of lesions, improvement of erythema, erosions, and maceration. Patient reported improvement.	None	Alzahrani et al^5^
50/M	5 y	Bilateral inguinal folds, perineal area	Topical corticosteroids, oral glycopyrrolate, acitretin, and naltrexone	25 mo	Betamethasone valerate cream	Significant improvement after 2 mo. Reduction in size and thickness of lesions, improvement of erythema, erosions, and maceration. Patient reported improvement.	Disease flare when treatment was interrupted but recaptured response when reintroduced	
70/M	33 y	Bilateral axillae, back of the neck	Numerous topical therapies, botulinum toxin injections, and corticosteroid injections	17 mo	Topical desonide lotion and antiperspirant	Significant improvement after 2 mo. Reduction in size and thickness of lesions, improvement of erythema, erosions, and maceration. Patient reported improvement.	None	
56/F	10 y	Trunk, extremities bilateral axillae, inguinal folds	Acitretin, antihistamines, apremilast, cyclosporine, doxycycline, dapsone, fluconazole, hydroxychloroquine, low-dose oral naltrexone, minocycline, methotrexate, oxybutynin, prednisone, sulfone, topical corticosteroids, and topical tacrolimus	14 mo	Topical clobetasol propionate ointment	Significant improvement after 2 mo of treatment. Reduction of 16 points in DLQI score and body surface area (50%) from baseline. PGA changed from 3 to 1 point.	None	Alamon-Reig et al^6^
52/M	12 y	Bilateral axillae and inguinal folds	Acitretin, apremilast, CO_2_ laser, doxycycline, low-dose oral naltrexone, minocycline, mycophenolate mofetil, oxybutynin, prednisone, and topical corticosteroids	13 mo	Topical fusidic acid, hydrocortisone ointment and oxybutynin 5 mg/d	No improvement of the patient’s reported DLQI. Fluctuating course over time.	None	
59/F	25 y	Trunk, inframammary folds, popliteal fossae, antecubital folds, vulva, and bilateral inguinal folds	Acitretin, antihistamines, apremilast, dapsone, low-dose oral naltrexone, minocycline, prednisone, and topical corticosteroids	16 mo	Antihistamines as needed	Significant improvement after 5 mo of treatment. Reduction of 16 points in DLQI score and body surface area (80%) from baseline. PGA changed from 2 to 1 point.	None	
57/F	Unspecified	Axillae, inframammary, and abdominal folds	Low-dose oral naltrexone	Unspecified	None	Improved pruritus and sleep after 1 week. Significant healing of the skin lesions was noticeable by week 12, with reduction in size and thickness of previous hyperkeratotic papules and erosions.	None	Brito Caldeira et al^7^
53/F	25 y	Bilateral axillae and inguinal folds	Topical erythromycin, oral antibiotics, intralesional and topical corticosteroids, and topical tacrolimus∗ (patient unable to tolerate oral apremilast caused by gastrointestinal side effects)	5 mo	Ruxolitinib cream	Significant improvement of axillae lesions after 2 wk of dupilumab monotherapy, lesions in inguinal folds persisted. Complete resolution of lesions after 1 mo of dupilumab + ruxolitinib cream dual therapy. At 5 mo follow-up, patient did not report any medication side effects or flare-ups of her HHD.	None	Khang et al^8^
22/F	Unspecified	Bilateral inframammary folds	Oral cyclosporine	4 mo	None	Considerable improvement in patient’s disease as supported by the disappearance of erythematous, erosive lesions after 4 mo of treatment. She has maintained this treatment response to dupilumab without any adverse effects.	None	Licata et al^9^
36/F	20+ y	Bilateral axillae	Calcipotriol ointment, topical clindamycin, oral cefuroxime, oral azithromycin, topical tacrolimus, IL-Tac injection, oral erythromycin, low-dose oral naltrexone, topical corticosteroids	10 mo	Low-dose oral naltrexone	She was initially started on dupilumab + low-dose oral naltrexone dual therapy and showed significant improvement after 7 wk of treatment. She attempted to discontinue naltrexone during this time, however, she flared up when doing so. At her 4 mo follow-up, she had been able to transition to dupilumab monotherapy and had resolution of her lesions.	None	∗

*DLQI*, Dermatology Life Quality Index; *F*, Female; *HHD*, Hailey-Hailey disease; *IL-Tac*, Intralesional triamcinolone; *M*, Male; *PGA*, Physician Global Assessment.

∗ Current patient being reported.

**Table II:** Timeline of treatment

Date	History/response to treatment	Treatment
10/8/2021	First visit	
	Responsive to as needed clobetasol	Clobetasol 0.05% ointment as needed
8/26/2022	Flaring after laser hair removal	
	Not responsive to clobetasol	Tacrolimus 0.1% ointment twice a day
9/14/2022	Not improved	
	Patient wanted biopsy confirmation	
9/27/2022	Biopsy confirmed	Calcipotriene 0.005% ointment twice a day
11/19/2022	Flaring—pregnant—malodor	Clindamycin 1% lotion twice a day
		Clobetasol 0.05% ointment twice a day
11/23/2022	Flaring—pregnant	Calcipotriene 0.005% ointment twice a day
		Clobetasol 0.05% ointment twice a day
1/12/2023	Flaring—pregnant	Erythromycin 250 mg
2/1/2023	Flaring—pregnant	IL-Tac 20 mg/cc (4 cc total)
		Chlorhexidine gluconate solution
		Petroleum jelly as needed
3/3/2023	Flaring—pregnant	Erythromycin 250 mg twice a day
		Clobetasol 0.05% ointment (with occlusion) twice a day
		Naltrexone 3 mg every day
4/5/2023	Flaring—pregnant—impetigo (she stopped erythromycin)	Erythromycin 250 mg twice a day × 1 mo
		Naltrexone 3 mg every day
		IL-Tac 20 mg/cc (3 cc per axilla)
4/20/2023	Flaring	Magnesium 300 mg every day
5/5/2023	Flaring with pain	Cefuroxime axetil 500 mg twice a day
		Lidocaine/prilocaine cream twice a day
		Erythromycin 250 mg stopped
5/9/2023	Much improved	
5/5/2023	Continues to improve	
6/14/2023	Flaring	Prior authorization dupilumab
6/21/2023	Flaring	Prednisone 40 mg every day × 2 wk
		TMP/SMX DS × 1 mo
7/10/2023	Flaring	Dupilumab injection 300 mg/mL
		Naltrexone 3 mg every day
7/12/2023	Rapid improvement	
7/19/2023	R > left axilla improvement	
	Impetigo	TMP/SMX DS twice a day × 2 mo
7/25/2023	Much improved	Dupilumab injection 300 mg/mL
		Naltrexone 3 mg every day
8/6/2023	Much improved	Dupilumab injection 300 mg/mL
		Naltrexone 3 mg every day
8/31/2023	Much improved	Dupilumab injection 300 mg/mL
		Naltrexone 3 mg every day
10/27/2023	Clear with dupilumab	Dupilumab injection 300 mg/mL
	Primary focal hyperhidrosis	OnabotulinumtoxinA 50 U/axilla
2/28/2024	Clear with dupilumab	Dupilumab injection 300 mg/mL
	Primary focal hyperhidrosis	OnabotulinumtoxinA 50 U/axilla

*IL-Tac*, Intralesional triamcinolone.

## Discussion

This is a case of a 36-year-old woman with a long-standing history of axillary HHD recalcitrant to many conventional treatments with a disease treatment course complicated by balancing efficacy with respect to safety for both the mother and fetus. Dupilumab, a monoclonal antibody targeting interleukin 4 and interleukin 13 receptors, is US Food and Drug Administration-approved for the treatment of a range of atopic conditions including atopic dermatitis, asthma, eosinophilic esophagitis, chronic rhinosinusitis with nasal polyposis, as well as for treatment of prurigo nodularis. Case reports and case series in the literature have reported successful off-label use of dupilumab for HHD (see Table I). The successful use of dupilumab in achieving disease remission may provide insight into the pathogenesis of disease, which remains poorly understood. As dupilumab targets T helper 2 (Th2) cytokines and has shown benefit in HHD, the role of the Th2 pathway in HHD must be considered. However, transcriptome profiling on lesional skin from a small group of patients with HHD demonstrated a Th17 inflammatory signature without evidence of a Th2 signature, and the mechanism of action of dupilumab in HHD remains unclear.^10^ Future controlled studies with transcriptome profiles are needed to better understand the role of dupilumab in the management of HHD and to further define pathways that lead to disease. Despite publication bias inherent in case reports favoring therapeutic success, dupilumab shows promise and warrants further exploration as a therapeutic option for a difficult disease.

## Conflicts of interest

None disclosed.
